# Brief Mindfulness Meditation Improves Attention in Novices: Evidence From ERPs and Moderation by Neuroticism

**DOI:** 10.3389/fnhum.2018.00315

**Published:** 2018-08-06

**Authors:** Catherine J. Norris, Daniel Creem, Reuben Hendler, Hedy Kober

**Affiliations:** ^1^Department of Psychology, Swarthmore College, Swarthmore, PA, United States; ^2^Psychiatry Department, Massachusetts General Hospital, Boston, MA, United States; ^3^Departments of Psychiatry and Psychology, Yale University, New Haven, CT, United States

**Keywords:** mindfulness meditation, neuroticism, attention, flanker, ANT, N2, P3b

## Abstract

Past research has found that mindfulness meditation training improves executive attention. Event-related potentials (ERPs) have indicated that this effect could be driven by more efficient allocation of resources on demanding attentional tasks, such as the Flanker Task and the Attention Network Test (ANT). However, it is not clear whether these changes depend on long-term practice. In two studies, we sought to investigate the effects of a brief, 10-min meditation session on attention in novice meditators, compared to a control activity. We also tested moderation by individual differences in neuroticism and the possible underlying neural mechanisms driving these effects, using ERPs. In Study 1, participants randomly assigned to listen to a 10-min meditation tape had better accuracy on incongruent trials on a Flanker task, with no detriment in reaction times (RTs), indicating better allocation of resources. In Study 2, those assigned to listen to a meditation tape performed an ANT more quickly than control participants, with no detriment in performance. Neuroticism moderated both of these effects, and ERPs showed that those individuals lower in neuroticism who meditated for 10 min exhibited a larger N2 to incongruent trials compared to those who listened to a control tape; whereas those individuals higher in neuroticism did not. Together, our results support the hypothesis that even brief meditation improves allocation of attentional resources in some novices.

## Introduction

Mindfulness may be used to describe a variety of practices and processes (e.g., van Dam et al., 2018); nevertheless, it is most often defined as a two-component process that includes: (1) attention to present moment experience, coupled with (2) an attitude that is open, non-reactive, and accepting of things as they are (Bishop et al., 2004; Ludwig and Kabat-Zinn, 2008; Kabat-Zinn, 2017). Over the past few decades, a wealth of research has emerged in both academic journals and popular media on the benefits of mindfulness meditation for attention (Sedlmeier et al., 2012), negative mood (Goyal et al., 2014), mental health (Hofmann et al., 2010), addictions (Brewer et al., 2011a; Chiesa and Serretti, 2014; Bowen et al., 2014), and many other factors (e.g., creativity; Ding et al., 2014). One premise in this area of research is that becoming mindful of an internal state or physiological function, such as one’s breath, can hone abilities such as focused attention, working memory, and acceptance. In turn, this is thought to have long-term positive consequences on attention, body awareness, emotion regulation, and perspectives on the self when mindfulness is trained and practiced over an extended period of time (e.g., Hölzel et al., 2011).

### Mindfulness Meditation Improves Attention

Much of the past research has focused on the effects of mindfulness meditation training on attentional processes, including alerting, orienting, and executive attention. Researchers have proposed that these three forms of attention are subserved by three separable neural networks (Posner and Petersen, 1990; Fan et al., 2002; Posner and Fan, 2008; Petersen and Posner, 2012). The alerting network maintains a state of vigilance or alertness and is measured as a readiness to attend to important or relevant stimuli when they arise. The orienting network is responsible for attending selectively to a sense modality or a location in space (Petersen and Posner, 2012) by prioritizing attention to a subset of possible inputs. The executive control network is responsible for deciding between competing inputs, and therefore plays an important role in conflict detection. Although these three networks clearly are all critical for attention, they are thought to function independently and are often measured separately.

Broadly, research suggests that mindfulness meditation training improves attention, although the specific types of attention have varied among studies. For example, MacLean et al. (2010) found that 3 months of intense meditation training can improve performance on tasks of perceptual discrimination and sustained visual attention. Elliott et al. (2014) showed that a weeklong intensive meditation retreat can improve both executive attention and alerting (but not orienting). Jha et al. (2007) examined alerting, orienting and executive attention in three samples: a control sample of meditation naïve participants who did not undergo an intervention, a sample of naïve meditators who completed an 8-week MBSR (i.e., mindfulness-based stress reduction) course, and a sample of experienced meditators who completed a 1-month intensive MBSR retreat. At Time 1 (i.e., before interventions), participants in the retreat group showed better executive attention than the other two groups; At Time 2 (after interventions), participants in the MBSR course showed better orienting and participants in the retreat group showed better alerting, both in comparison to the other two groups. Notably, although there was no group difference found on conflict monitoring at Time 2, the authors did not conduct an analysis on change scores for each attentional network. Thus, it is possible that both the non-intervention group and the course group showed improved conflict monitoring/executive attention at Time 2 compared to Time 1. Tang et al. (2007) used a slightly less time-intensive approach and reported that 5 days of 20-min training sessions can improve executive attention. In a review article comparing multiple forms of meditation, Lippelt et al. (2014) conclude that there is good evidence to suggest that focused attention meditation (FAM; such as mindfulness) increases sustained attention (Carter et al., 2005; Brefczynski-Lewis et al., 2007). Notably, all of these studies have utilized relatively-extensive meditation training, including multiple training sessions administered over an extended period of time. Furthermore, any differences observed between the naïve meditators (and an 8-week MBSR course) and the experienced meditators (and a 1-month MBSR retreat; Jha et al., 2007) may be attributable either to differences in previous experience or to differences between the MBSR trainings.

Indeed, studies on the effects of mindfulness meditation most often involve either an extended immersive experience (e.g., a 3-month retreat) or repeated daily practice, either in the form of a multi-week course, or days, weeks, or months of self-guided meditation. Specifically, the vast majority of published work has tested the effects of eight sessions of training or longer (e.g., Hofmann et al., 2010; Brewer et al., 2011b), and, although these studies have often documented beneficial outcomes of mindfulness meditation practice, the relevance of such time-consuming, extensive training is debatable for individuals who may be unmotivated or unable to dedicate the time and resources necessary to reap such benefits. This can be framed as a question of “dose”—once someone begins to practice mindfulness, how soon can they expect to see beneficial effects (e.g., Tang et al., 2015; Zeidan, 2015)? A few recent studies have shown that 3–4 days of training are associated with some beneficial effects (Zeidan et al., 2010a,b,c).

Although most research on the effects of mindfulness meditation on cognition has involved more complicated, long-term training (either utilizing multiple sessions or full immersion), it is worth noting that a handful of studies have examined the impact of a more brief meditation intervention^1^. For example, Wenk-Sormaz (2005) found that 20 min of transcendental meditation improved performance on the Stroop task (Study 1) and reduced habitual responding on a category production task (Study 2), compared to two control conditions—a cognitive control task (Study 1: mnemonic learning; Study 2: a timed general knowledge test) and a resting control (in which participants simply let their minds wander for 20 min). In Study 1, however, participants completed three sessions in the laboratory, the first two of which incorporated the practice of their randomly-assigned condition. Furthermore, the control conditions utilized in both studies were arguably not well matched to the 20-min meditation and allow for a number of confounds (e.g., visual and auditory input, cognitive effort) that complicate interpretation of their results. In a study more closely related to our current work, Larson et al. (2013) randomly assigned non-meditators to listen to either mindfulness or control audio clips, which were well-matched in voice and duration (~14 min each), and examined subsequent performance on a Flanker task (Eriksen and Eriksen, 1974). Their results indicated no behavioral differences between experimental groups on the Flanker task, although they did report reliable differences in psychophysiological measures (e.g., blood pressure, event-related brain potentials [ERPs]). Similarly, Johnson et al. (2015) examined the effects of a single 25-min mindfulness meditation (vs. a sham meditation and a control condition in which participants listened to a book on tape) on mood and a series of cognitive tasks testing working memory, memory span, attentional shifting and visual tracking. They found no effects of meditation on any measure of cognitive performance.

Researchers have also begun to compare different forms of meditation to more specifically investigate the effects of meditation on attention. Colzato et al. (2016) had participants complete two experimental sessions: one in which they underwent 17 min of FAM, which requires attention to a chosen object and is thought to increase top-down cognitive control, and a second in which they underwent open monitoring meditation (OMM), which requires open monitoring of experience and is thought to decrease top-down cognitive control (session order was counterbalanced). Although no differences were found between the two conditions on a measure of attentional focusing, following OMM participants showed greater failure to suppress task-irrelevant information. In a separate study that utilized a between-participants design, participants who completed OMM exhibited a smaller attentional blink, indicating more efficient allocation of attention over time, than did those who completed FAM (Colzato et al., 2015). Thus, different forms of meditation may have differential effects of attention.

In sum, research on the effects of a single, brief session of mindfulness meditation on cognitive performance is extremely rare and has produced few reliable findings. Thus, across two studies, we focus on meditation-naïve college students, to examine whether 10 min of mindfulness meditation instruction may have an immediate impact on attention, as compared to a control group matched in age, gender, previous experience and other interpersonal factors (e.g., personality). As an exploratory aim, we also tested whether individual differences in neuroticism, which is characterized by anxiety, high negative affect and worry, may moderate the effects of brief mindfulness meditation instruction. We focus on meditation-naïve participants as a form of *tabula rasa* to minimize the impact of any past experience with meditation on our measures of interest. We employ a single, short mindfulness meditation instruction period for two important reasons. First, we modeled the instructions after the foundational introductory mindfulness instructions offered by Kabat-Zinn (2006) as used in MBSR courses to explore its potential impact. Further, we chose this short, 10-min mindfulness meditation session as it would likely be more feasible, tenable, and both more cost- and time-effective for members of the general population than a longer, more extensive intervention. This will also allow us to explore minimum “dose” effects, as discussed above. Further, we explore the effects of neuroticism, a well-studied personality variable, to investigate the possibility that pre-existing individual differences may affect the efficacy of brief interventions for meditation-naïve individuals. As such, this novel approach represents a test of the impact of one’s first exposure to mindfulness meditation on attention and may greatly expand our knowledge of the power of meditation, its boundary conditions, as well as its potential for practice in daily life.

A second exploratory aim of the current studies was to investigate the potential neural mechanisms underlying the effects of mindfulness meditation on attention using ERPs. The study of ERPs allows us to investigate the specific neural components or processes that may be affected by meditation, as they unfold over time. N2—an anterior negative component thought to index detection of stimulus mismatch (Luck, 2012), response competition (Nieuwenhuis et al., 2003), and cognitive control (Folstein and Van Petten, 2008)—is one such component that has previously been shown to be impacted by meditation (van Leeuwen et al., 2012), and which is often implicated in studies requiring attention (e.g., Flanker tasks; Kopp et al., 1996b; Heil et al., 2000). Moore et al. (2012) reported that, following mindfulness meditation training, individuals exhibited larger N2 amplitudes on an attention task, indicating improved attentional control. In addition, the P3b component—a posterior positive component associated with attention allocation (Polich, 2007)—has also been implicated in studies of the effects of meditation on attention. Experienced meditators exhibit a larger P3b to an oddball stimulus following meditation (Delgado-Pastor et al., 2013) and a smaller P3b to the first target on an attentional-blink task, indicating more efficient attention allocation (Slagter et al., 2007). Thus, in Study 2, we used ERPs to further probe the effects of a brief mindfulness session on attention in novice meditators, specifically on these two ERP components.

In sum, we hypothesized that a brief mindfulness meditation would improve executive attention even in meditation-naïve participants. Based on the vast literature on neuroticism (for some relevant examples see Gunthert et al., 1999; Schneider, 2004), we propose that individuals higher in neuroticism may be less likely to reap the benefits of meditation, due perhaps to an inability or unwillingness to follow the meditation instructions, and we examined ERPs—specifically the N2 and P3b components, which are implicated in attention control and allocation—to better understand the neural correlates of the relationship between brief mindfulness meditation, executive attention, and individual differences in neuroticism.

## Study 1

In an initial attempt to examine the effects of brief meditation on attention in novice meditators, we asked participants to listen to a 10-min audio tape: mindfulness meditation vs. control. After listening to the tape, participants completed a version of the Flanker task (Eriksen and Eriksen, 1974; Eriksen, 1995; Larson et al., 2013), a measure of executive attentional control (Fan et al., 2002; Posner and Fan, 2008). Participants also completed the Big 5 Personality Dimension Inventory to allow for the investigation of moderation by individual differences in neuroticism. Thus, our hypotheses for Study 1 were:

Hypothesis 1a: a brief mindfulness meditation will improve executive attention (either in better accuracy or reduced response times (RTs) for incongruent trials) in meditation-naïve participants, compared to a control condition.

Hypothesis 1b: individual differences in neuroticism will moderate this effect, such that individuals higher in neuroticism will not show as strong an improvement in executive attention following a brief mindfulness meditation as those lower in neuroticism.

## Method

### Participants

Forty undergraduate students (14 female) between the ages of 17 and 22 (*M* = 19.48, *SD* = 1.18) were recruited from Swarthmore College. Three participants were omitted from final analyses because their average raw accuracy and/or RTs were greater than 3 SDs from the mean (i.e., outliers), leaving a final sample size of 37 (12 female; *M*_age_ = 19.51, *SD* = 1.19). Participants were entered into a raffle for one of two $25 prizes as compensation for completion of the study. This study was carried out in accordance with the recommendations of Swarthmore College Institutional Review Board with written informed consent from all subjects. All subjects gave written informed consent in accordance with the Declaration of Helsinki. The protocol was approved by the Swarthmore College IRB.

### Procedure

**Figure 1:**
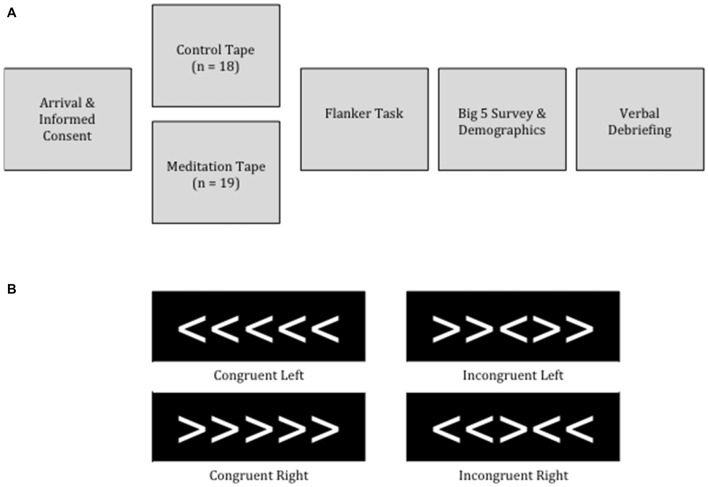
**(A)** Session timeline. **(B)** Trial types for the Flanker Task used in Study 1.

Upon arriving at the laboratory, participants were told that the purpose of the study was to investigate the effects of auditory attention on visual acuity (see Figure 1A for schematic representation of the session) and gave written and oral informed consent. Each participant was seated in front of a desktop computer and was asked to wear headphones and a blindfold, to allow them to focus on the audio tape. After participants completed listening to the 10-min tape, the experimenter returned, removed the blindfold and headphones and provided verbal instructions for the Flanker task. Participants completed 12 practice trials and were given the opportunity to ask questions before beginning the experimental Flanker trials. Following the Flanker task, participants completed the Big 5 Personality Inventory (John et al., 1991) and a demographic survey. Finally, the experimenter and participant engaged in a face-to-face funneled debriefing interview.

### Experimental Conditions

Participants were randomly assigned to either listen to a 10-min guided meditation tape (meditation) or a 10-min audio control tape (control). The mindfulness meditation tape was developed based on classic mindfulness instructions used in MBSR, on several typical definitions of mindfulness (e.g., Bishop et al., 2004; Kabat-Zinn, 2017; van Dam et al., 2018) and in consultation with several Vipassana meditation teachers. This tape led participants through a breath-focused mindfulness exercise oriented towards beginners. It included instructions such as “please set the intention to observe your experience with an accepting attitude,” “please notice and begin to follow the natural and spontaneous movement of the breath, not trying to change it in any way,” and “stay open and curious about your experience.” As such, the instructions-oriented participants towards “the awareness that arises from paying attention, on purpose, in the present moment, and non-judgmentally” (Kabat-Zinn, 2017). In this way, it closely modeled what participants might be doing in their first session of an MBSR course, and after several typical definitions of mindfulness that include a component of attention oriented to the present moment, coupled with an attitude that is open-hearted and accepting (Bishop et al., 2004; Ludwig and Kabat-Zinn, 2008; Kabat-Zinn, 2017). The control tape was a reading of a National Geographic article about giant sequoias. Importantly, both tapes were recorded by the same person, used the same speed of speech, and featured the same number of words, with similar word frequencies. In addition, both tapes began with instructions on posture within the first few seconds and included pauses at approximately the same times and for similar durations, throughout.

### Flanker Task

The flanker task was delivered using E-Prime 2.0 software on a Dell computer with a 22″ LCD monitor (refresh rate = 60 Hz). The flanker array consisted of white arrowheads on a black background and was 4.5 cm wide by 1.3 cm high. On average, participants sat approximately 70 cm from the screen, producing a visual angle of the array width of 0.026 degrees and of the array height of 0.091 degrees. Each trial consisted of a 500 ms white fixation cross in the center of the black screen, followed by an array of five arrows, which remained on the center of the screen until a response was made (Figure 1B). Participants pressed the “f” key with their left hand if the center arrow was facing left, and the “j” key with their right hand if the center arrow was facing right. Flanking arrows were facing either in the same direction (i.e., congruent trials), or in the opposite direction (incongruent trials; Figure 1B). There were 20 trials in each cell of the 2 (direction: left, right) × 2 (trial type: congruent, incongruent) design, resulting in a total of 80 trials, presented randomly. Participants were told to respond as quickly and accurately as possible. As soon as a response was made, the next trial began (i.e., there was no intertrial interval); this decision was made to decrease the length of the experiment.

### Big 5 Personality Inventory

After the flanker task, participants completed the Big 5 Personality Inventory (John et al., 1991), a self-report survey consisting of 44 items designed to measure five personality factors: Openness, Conscientiousness, Extraversion, Agreeableness and Neuroticism. Participants indicated the degree to which they agreed or disagreed with each item on a 5-point Likert scale, with endpoints labeled *disagree strongly* (1) and *agree strongly* (5). Each item began with the phrase “I see myself as someone who…”; sample items for the neuroticism subscale items include: “worries a lot” and “is emotionally stable, not easily upset,” with the latter reverse-coded. Responses to individual items were averaged separately for each factor, and averages were *z*-scored before further analysis for ease of interpretation.

### Demographic Survey

Participants also completed a standard demographic survey in which they reported their age, gender (male, female), race and ethnicity.

### Debriefing

Finally, participants completed a funneled debriefing interview in which they were given the opportunity to report any suspicion about the true purpose of the study, as well as reporting any previous experience with meditation, including duration and frequency of practice. An experimenter blind to tape condition coded the verbal responses regarding meditation experience on a 5-point scale, with 0 indicating no previous experience with meditation, and four indicating daily practice, extensive training and/or attendance at multiple retreats.

## Results^2^

We conducted independent samples *t*-tests to examine any group differences between participants randomly assigned to listen to the meditation tape vs. those randomly assigned to listen to the control tape on variables including: age, gender, race, Big 5 Personality traits and meditation experience (Table 1). There were no significant group differences on any of these measures.

**Table 1:** Means (SDs) for participants randomly assigned to listen to the meditation tape and the control tape in Study 1.

	Meditation tape	Control tape	*t*-statistic
*N*	18	19
Age	19.22 (1.17)	19.79 (1.18)	1.47 (*p* = 0.15)
Gender (M = 0; F = 1)	0.39 (0.50)	0.26 (0.45)	0.80 (*p* = 0.43)
Race (White = 0; Non-White = 1)	0.39 (0.50)	0.28 (0.46)	0.69 (*p* = 0.49)
Big 5 Personality Traits (z-scored)			
Agreeableness	−0.14 (1.01)	0.13 (1.00)	0.82 (*p* = 0.42)
Conscientiousness	−0.02 (0.83)	0.02 (1.16)	0.13 (*p* = 0.90)
Extraversion	−0.05 (1.09)	0.05 (0.93)	0.30 (*p* = 0.77)
Neuroticism	0.03 (0.97)	−0.01 (1.05)	0.14 (*p* = 0.89)
Openness	−0.03 (0.99)	0.03 (1.03)	0.16 (*p* = 0.87)
Meditation experience*	0.67 (0.77)	0.58 (0.61)	0.39 (*p* = 0.70)

*An experimenter coded participants’ verbal descriptions of their experience with meditation on a scale from no experience (0) to regular practice (4).

### Response Times

RTs for trials on which participants responded correctly were subjected to a 2 (condition: meditation, control) × 2 (trial type: congruent, incongruent) general linear model (GLM), with the first factor manipulated between-participants and the second factor manipulated within-participants (collapsing across arrow direction). The main effect of trial type (*F*_(1,35)_ = 129.32, *p* < 0.001, $\eta_{p}^{2}$ = 0.79), indicated that participants were faster to respond on congruent trials (*M* = 427.05 ms, *SE* = 6.84) than on incongruent trials (*M* = 466.26, *SE* = 7.53), replicating a wealth of prior research. No other effects reached traditional levels of significance (i.e., *p* < 0.05).

### Accuracy

**Figure 2:**
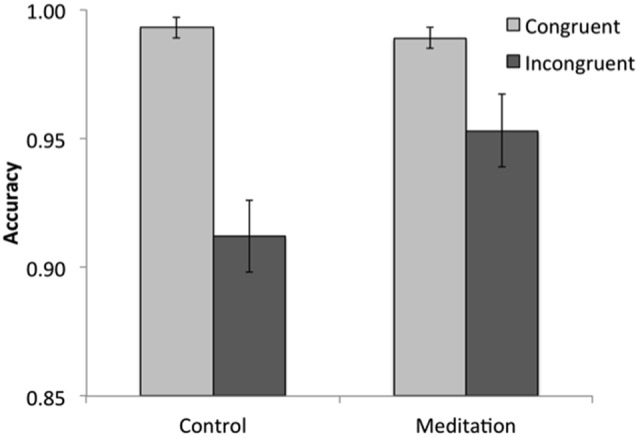
The interaction between condition and trial type in Study 1. Both groups were more accurate on congruent than on incongruent trials, but individuals in the meditation condition performed better on incongruent trials than did those in the control condition.

Proportions of correct trials (i.e., accuracy) were subjected to a similar 2 (condition: meditation, control) × 2 (trial type: congruent, incongruent) GLM. The main effect of trial type (*F*_(1,35)_ = 35.12, *p* < 0.001, $\eta_{p}^{2}$ = 0.50) indicated that participants were more accurate on congruent (*M* = 0.99, *SE* = 0.003) than on incongruent (*M* = 0.93, *SE* = 0.01) trials, another replication of past research. The main effect of condition was marginally significant (*F*_(1,35)_ = 3.10, *p* = 0.087, $\eta_{p}^{2}$ = 0.08), indicating that participants in the meditation condition were more accurate (*M* = 0.97, *SE* = 0.007) than participants in the control condition (*M* = 0.95, *SE* = 0.007). Further, there was a significant interaction between trial type and condition (*F*_(1,35)_ = 5.24, *p* = 0.028, $\eta_{p}^{2}$ = 0.13). Pairwise tests showed that both groups of participants were more accurate on congruent than on incongruent trials (*p*s < 0.05). More critically, pairwise tests showed that whereas participants in the meditation condition (*M* = 0.99, *SE* = 0.004) and the control condition (*M* = 0.99, *SE* = 0.004) performed equally well on congruent trials (*p* = 0.392), participants in the brief meditation condition performed significantly better on incongruent trials (*M* = 0.95, *SE* = 0.01) than did those in the control condition (*M* = 0.91, *SE* = 0.01), *p* = 0.044 (Figure 2).

### Flanker Effect Scores

To further probe the effects of brief meditation on attention on the Flanker task, we calculated difference scores to capture the “Flanker effect” in RTs on correct trials (incongruent—congruent) and accuracy (congruent—incongruent), separately. Two independent samples *t*-tests conducted on these difference scores showed no difference between meditation and control conditions in correct RTs (*p* = 0.483) but did show a significant difference in accuracy (*t*_(35)_ = 2.29, *p* = 0.028), such that participants in the meditation condition (*M* = 0.04, *SE* = 0.06) exhibited a smaller Flanker effect than those in the control condition (*M* = 0.08, *SE* = 0.06). Thus, participants in the meditation condition showed a smaller Flanker effect in accuracy—reflecting better executive attentional control—as compared to those in the control condition, driven by better performance on incongruent trials. Thus, the accuracy results provide support for Hypothesis 1a.

### Moderation by Neuroticism

First, we conducted an independent samples *t*-test to examine whether neuroticism differed between conditions, despite random assignment to condition (i.e., meditation vs. control tape). As expected, neuroticism did not differ between participants assigned to the brief meditation tape (*M* = 0.03, *SD* = 0.97) and those assigned to the control tape (*M* = −0.02, *SD* = 1.06; *t*_(35)_ = 0.14, *p* = 0.889). Thus, differences in task performance between conditions could not be attributed to individual differences in neuroticism.

In order to investigate moderation by neuroticism, we used neuroticism as a continuous variable rather than subdivide participants into smaller groups (e.g., median splits, extreme groups). This latter approach would be inappropriate for a number of reasons, including our relatively small sample size and the fact that median splits are generally not appropriate for measures that are truly normally distributed (such as neuroticism in the current study). In summarizing the issues with median splits, Aiken and West (1991, p. 4) state that: “Median splits of continuous variables throw away information, reducing the power of the statistical test: they make it much more difficult to detect significant effects when in fact they do exist (Cohen, 1983).” Similar arguments have been made by Maxwell and Delaney (1993) and MacCallum et al. (2002). Thus, neuroticism was entered as a continuous variable, which utilizes every score and allows the investigation of both main effects and interactions involving neuroticism. When an effect involving neuroticism emerged, we examined estimates 1 SD above and below the mean to interpret that effect (i.e., parameter estimation). This is a standard analysis used in individual differences research in psychology (Aiken and West, 1991; Norris et al., 2007, 2011; Rutherford, 2011).

To examine moderation of the effects of meditation on attention by individual differences in neuroticism, RTs were subjected to a 2 (condition: meditation, control) × 2 (trial type: congruent, incongruent) × *z*-scored neuroticism GLM, with the first factor manipulated between-participants, the second factor manipulated within-participants, and neuroticism entered as a continuous between-participants covariate. This analysis allows for the examination of main effects and the interaction between condition and trial time holding neuroticism constant, as well as investigating the main effect of neuroticism and its interactions with all other variables. This analysis revealed a main effect of trial type (*F*_(1,33)_ = 126.30, *p* < 0.001, $\eta_{p}^{2}$ = 0.79), such that participants were faster on congruent than on incongruent trials even when controlling for neuroticism.

**Figure 3:**
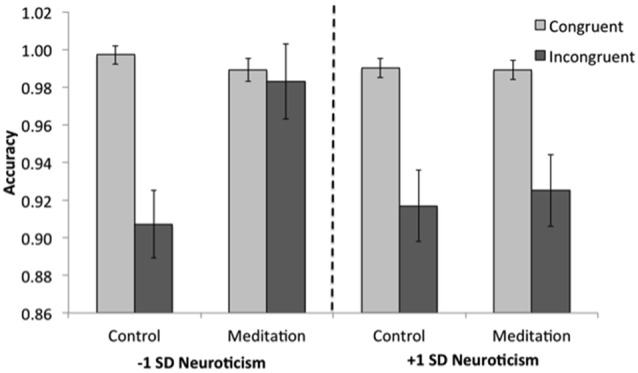
The interaction between condition, trial type and neuroticism in Study 1. Meditation was effective in improving performance on incongruent trials for individuals lower in neuroticism (−1 SD) but not for those higher in neuroticism (+1 SD).

A similar GLM was conducted on accuracy scores. As expected, the main and interaction effects reported above held when controlling for neuroticism, including the condition × trial type interaction, indicating that individuals in the brief meditation condition were more accurate on incongruent trials than were those in the control condition. However, we also found a marginal condition × trial type × neuroticism interaction (*F*_(1,33)_ = 3.72, *p* = 0.062, $\eta_{p}^{2}$ = 0.10). To better understand this interaction, we examined accuracy estimates at 1 SD above and below the mean neuroticism score; this is standard parameter estimation for linear models (Aiken and West, 1991; Rutherford, 2011). Those individuals lower in neuroticism (−1 SD) generally exhibited the previously-reported pattern: individuals in the control condition were more accurate on congruent than incongruent trials (*p* < 0.001), the two groups did not differ in their accuracies on congruent trials (*p* = 0.302), but individuals in the brief meditation condition performed better on incongruent trials (*M* = 0.98, *SE* = 0.02) than did those in the control condition (*M* = 0.91, *SE* = 0.02; *p* = 0.009, see Figure 3). Indeed, brief meditation improved performance to such a degree that lower neuroticism participants in the brief meditation condition performed as well on incongruent trials (*M* = 0.98, *SE* = 0.02) as they did on congruent trials (*M* = 0.99, *SE* = 0.01), *p* = 0.780. Individuals higher in neuroticism (+1 SD), however, showed no effect of meditation: both groups were more accurate on congruent than on incongruent trials (*p*s < 0.005) and did not differ in accuracy on either trial type (*p*s > 0.750; Figure 3). Thus, the accuracy results provide support for Hypothesis 1b.

## Discussion

Results from Study 1 suggest that a brief 10-min guided mindfulness meditation instruction period can improve executive attentional control even in naïve, inexperienced meditators (Hypothesis 1a). This is a novel and important finding, suggesting that individuals who are merely initiating a meditation practice may reap benefits after a single brief session. As such, it redefines the boundary conditions of the efficacy of meditation practice, which has predominantly been studied with longer courses of meditation training. Interestingly, this meditation-induced improvement in performance was most pronounced in individuals lower in neuroticism; individuals higher in neuroticism did not exhibit any performance boost following meditation (Hypothesis 1b). Neuroticism may thus prevent individuals from reaping the benefits of an initial, brief meditation. Importantly, we do not make any inferences about the quality of the state experienced by participant in the mindfulness meditation condition and acknowledge that it may differ from a mindful state that can be achieved after a longer meditation practice. Nevertheless, the results show that following typical mindfulness meditation instructions—similar to those that begin any MBSR course—has a significant effect on performance.

## Study 2

Given the novelty of the findings, we sought to conceptually replicate results from Study 1 in a new sample and using a different albeit related task. Thus, in Study 2 we used the Attention Network Task (ANT; Fan et al., 2002; see “Study 2 Method” section for a full description). The ANT was chosen for a number of reasons. First, the ANT includes a traditional Flanker task embedded in an attention-cuing task. Thus, the ANT allowed for a conceptual replication (rather than a direct replication, thereby increasing reliability) of the results from Study 1. In addition, the attentional cues allow for the exploration of other attention systems (i.e., alerting, orienting; however, these analyses are beyond the scope of the current article). Finally, the ANT has previously been used extensively with electrocortical measures in the past (e.g., Neuhaus et al., 2010); we wanted to use a task that would allow us to investigate neural correlates of the effects of meditation on attention. We employed the ANT to allow for a replication of results from Study 1; specifically, that following a brief meditation tape, participants would show better performance on a test of executive control. Thus, we focus on measures related to the executive control network (i.e., responses to congruent and incongruent flanker trials), as they are the most directly related to results from Study 1. Further, we collected event-related brain potentials (ERPs) to allow for a preliminary investigation of the neural mechanism(s) underlying the effects of meditation on attention. In particular, we predicted that the frontal N2, an ERP component enhanced during shifts in attention (Hietanen et al., 2008), response competition (Nieuwenhuis et al., 2003), and attentional control (Folstein and Van Petten, 2008); and commonly observed on Flanker tasks (Kopp et al., 1996b; Heil et al., 2000), may be impacted by brief meditation and may subsequently affect executive attentional control as measured on the ANT. Specifically, we predicted that participants who listened to the meditation tape would exhibit an enhanced (i.e., more negative) N2 as compared to those who listened to the control tape, especially on incongruent trials. Furthermore, the posterior P3b, a component associated with attention allocation (Polich, 2007), has also previously been shown to be impacted by meditation in experienced practitioners (Delgado-Pastor et al., 2013) and following extensive training (Slagter et al., 2007). We predicted that the P3b may also be impacted by brief meditation, such that participants who listened to the meditation tape would exhibit an enhanced (i.e., more positive) P3b as compared to those who listened to the control tape, especially on incongruent trials. These two findings would support our hypothesis that brief meditation may improve both conflict detection (N2) and allocation of attentional resources (P3b) on the ANT.

Furthermore, we sought to examine whether individual differences in neuroticism might moderate these effects. Given our results from Study 1, neuroticism may prevent meditation-naïve individuals from reaping the benefits of a brief meditation.

The primary aims of Study 2 were: (a) to provide a conceptual replication of results from Study 1 showing that a brief meditation can improve attention, particularly for individuals lower in neuroticism, and (b) to explore the possible neural mechanism(s) underlying these effects. To do this, we used the same general design from Study 1, except that participants completed the ANT instead of the Flanker task to allow for a conceptual replication of Study 1, and we collected continuous electroencephalography (EEG) during the procedure. Thus, our hypotheses for Study 2 were:

Hypothesis 2a (conceptual replication): a brief mindfulness meditation will improve executive attention (either in better accuracy or reduced RTs for incongruent trials) in meditation-naïve participants, compared to a control condition.

Hypothesis 2b (conceptual replication): individual differences in neuroticism will moderate this effect, such that individuals higher in neuroticism will not show as strong an improvement in executive attention following a brief mindfulness meditation as those lower in neuroticism.

Hypothesis 3: attention-related ERP components, specifically the N2 and the P3b, will reflect the interaction between neuroticism and executive attention, such that individuals lower in neuroticism will show a greater improvement in attention toward incongruent vs. congruent stimuli (as we found in Study 1), whereas individuals higher in neuroticism will not show this improvement.

## Method

### Participants

Fifty-nine undergraduate students (29 female) between the ages of 18 and 22 (*M* = 19.56, *SD* = 1.13) were recruited from Swarthmore College. Three participants were omitted from final analyses because their average raw accuracy and/or RTs were greater than 3 SDs from the mean (i.e., outliers), leaving a final sample size of 56 (27 female; *M*_age_ = 19.52, *SD* = 1.14). Participants either were paid $15 or received course credit as compensation for completion of the study. This study was carried out in accordance with the recommendations of Swarthmore College Institutional Review Board with written informed consent from all subjects. All subjects gave written informed consent in accordance with the Declaration of Helsinki. The protocol was approved by the Swarthmore College IRB.

### Procedure

Similarly to Study 1, upon arriving at the ERP laboratory at Swarthmore College, participants were told that they would be listening to a tape and wearing a blindfold to minimize distraction. After providing informed consent, they were seated in front of a desktop computer and were given oral and visual instructions on how to complete the ANT (Fan et al., 2002). Participants completed 24 practice trials with feedback and were given the opportunity to ask clarification questions about the task before continuing. Instructions and practice trials occurred before the experimental manipulation to guarantee that any observed effects on the ANT were due directly to the manipulation rather than any effect of the manipulation on task learning. Following the practice ANT, an electrode net was applied for the collection of continuous EEG (see below for details). Similarly to Study 1, participants were blindfolded and the experimenter left the room while participants listened to the audio tape, which was delivered through two Logitech desktop computer speakers. After the tape, the experimenter returned, removed the participant’s blindfold, and verified that the participant remembered how to perform the ANT (all did). The participant then completed the entirety of the ANT (see below for details). Following completion of the ANT, the experimenter removed the electrode net and participants completed the Big 5 Personality Inventory (Goldberg, 1992) and a standard demographics survey (see Study 1).

### Experimental Conditions

As in Study 1, participants were randomly assigned to listen to either a 10-min guided audio meditation tape (meditation) or a 10-min audio control tape (control).

### Attention Network Test (ANT)

**Figure 4:**
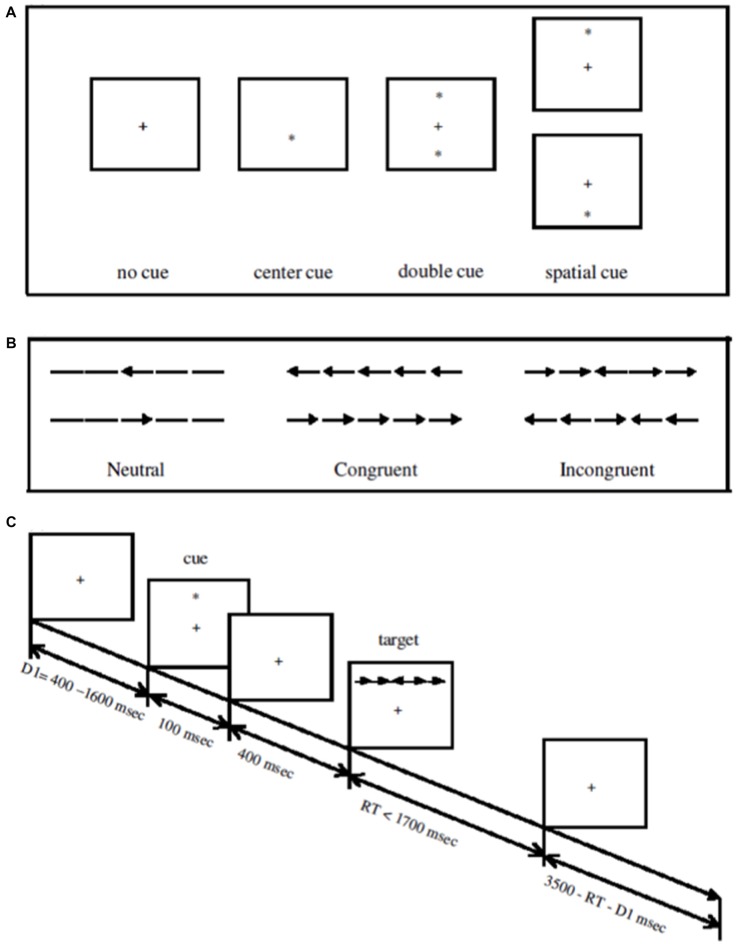
Trial structure for the Attention Network Task used in Study 2 (Figure 1 from Fan et al., 2002, used with permission from MIT Press). **(A)** Cue types. **(B)** Flanker conditions. **(C)** Schematic of trial structure.

The ANT was programed in E-prime version 2.0 according to the description in Fan et al. (2002), and also referencing the description in Neuhaus et al. (2010). The ANT differs from the Flanker task as utilized in Study 1 in a number of ways. First, the ANT includes three trial types: in addition to congruent (i.e., flanking arrows facing the same direction as the target central arrow) and incongruent trials (flanking arrows facing the opposite direction as the target central arrow), the ANT also measures responses to neutral trials, in which the target central arrow is flanked by dashes instead of arrows (Figure 4). Second, the ANT displays the Flanker array either above or below a central fixation cross. Third, the ANT includes an additional cueing factor, in which the appearance of the Flanker array is preceded by a central cue (i.e., an asterisk presented in the position of the fixation cross), a double cue (two asterisks appearing above and below the fixation cross), a spatial cue (one asterisk appearing either above or below the fixation cross), or by no cue. Spatial cues always indicated the location of the Flanker array. These differences allow for the calculation of different types of attention, including alerting, orienting, and executive control (Fan et al., 2002). Note that given our primary interest in replicating results from Study 1, as well as research suggesting that the three attentional networks (i.e., alerting, orienting, executive control) are functionally integrated and may interact (Fan et al., 2002), we focus solely on the functioning of the executive control network (i.e., responses to the congruent and incongruent Flanker arrays).

A fixation cross was presented in the center of the screen throughout the experiment. Each trial began with a randomly determined 400–1600 ms fixation period (see Figure 4 for trial schematics). Next, a cue appeared (except on no cue trials) for 100 ms, followed by an additional 400 ms of fixation. Then, the Flanker array appeared, either above or below the fixation cross. The Flanker array remained on the screen until the participant responded or until 1700 ms had passed, whichever was of shorter duration. After the Flanker array, a period of fixation was generated such that the total duration of each trial was 3500 ms. 24 repetitions of each of 3 (trial type: congruent, incongruent, neutral) × 4 (cue: no cue, central cue, double cue, spatial cue) conditions were presented for a total of 288 trials, split into three blocks of 96 trials each. Participants were given breaks between blocks to rest.

### Big 5 Personality Inventory

Following the ANT, the electrode net was removed and participants completed the Big 5 Personality Inventory (Goldberg, 1992), a self-report survey consisting of 100 items designed to measure five personality factors (20 items per factor): Agreeableness, Conscientiousness, Intellect, Emotional Stability (i.e., the inverse of Neuroticism), and Surgency (i.e., Extraversion). Instructions guided participants to: “Please use the following list of common human traits to describe yourself as accurately as possible. Describe yourself as you see yourself at the present time, not as you wish to be in the future. Describe yourself as you are generally or typically, as compared with other persons you know of the same sex and of roughly the same age. For each trait, please click on the box that most accurately describes you.” Participants responded to each item on a 9-point Likert scale, with endpoints labeled *extremely inaccurate*: (1) and *extremely accurate* (9); all nine scale points were labeled. We were primarily interested in the Emotional Stability subscale (i.e., the inverse of neuroticism): sample items included “Anxious” and “Imperturbable” (the latter is reverse coded such that higher responses indicated greater neuroticism). Responses were averaged across all 20 items and *z*-scored for inclusion in analyses. It is worth noting that we did use different scales to measure neuroticism in Study 1 and Study 2. This was primarily due to time constraints in Study 1; the scale used in Study 2 (Goldberg, 1992) contains more items, and may therefore have better reliability. Although most measures of neuroticism are highly correlated, we mention the use of different scales as it may explain any potential differences in results.

### Debriefing

Finally, participants completed a funneled debriefing interview similar to that from Study 1.

## Electroencephalography Data Collection and Analysis

EEG was recorded using a 64-channel Electrical Geodesics Incorporated (EGI) Geodesic Sensor Net (Electrical Geodesics Inc.^3^) which consists of electrodes embedded in small sponges and connected via a web of elastomer bands that fits like a cap. Before each experimental session, the net was soaked in a saline solution for 5 min to saturate the sponges with a conductive fluid and allow for the recording of EEG from the scalp without direct contact. The sensor net was connected to an EGI Net Amps 300 electrical amplifier, which then fed into a Mac computer running NetStation 4.5.4 software. The online sampling rate was 1000 Hz.

### Placement and Impedances

The net was placed on the scalp according to guidelines established by EGI Inc. EGI uses a Cartesian coordinate system with X, Y and Z dimensions to specify sensor positions in 3D space on the scalp surface; the 10-10 International System equivalents have been established previously to simplify comparisons across studies (Luu and Ferree, 2005). Position of the net was checked to verify proper location of sensors before the experimenter tested signal impedances. We attempted to keep impedances of all electrodes <40 kΩ; if this goal was not obtained within 20 min of preparation, the experiment began regardless. Impedances were checked and fixed during breaks, although contact with participants was kept minimal to minimize potential interference with the manipulation.

### Data Reduction

After data collection, raw EEG was filtered with a 0.1 Hz high-pass and a 30 Hz low-pass filter, and down-sampled to 250 samples/s. The continuous EEG was segmented based on the time at which the Flanker array appeared in each trial; the segment window was 800 ms pre-Flanker to 700 ms post-Flanker. Segments were subjected to a baseline correction to the first 200 ms of the epoch (i.e., the initial fixation cross) and then were run through an automatic artifact detection tool. A channel was marked bad within a segment if: (a) there was *a* > 75 μV change within the window; or (b) there was *a* < 2 μV change within the window. If a channel was marked bad for more than 20% of trials, it was marked bad throughout. A segment was marked bad if (a) it had >10 bad channels; (b) it had a blink (defined as a spike in the eye channels larger than 140 μV); or (c) it had an eye movement (defined as a spike in the eye-movement channels larger than 55 μV). The segments were then run through an automatic bad channel replacement tool, which replaced the bad channels with interpolated data from surrounding channels using spherical splines. After this, the segments were run through an automatic ocular artifact removal tool with a blink slope threshold of 14 μV/ms. If an ocular artifact was detected within a segment, it was statistically removed (see www.egi.com for more information; Gratton et al., 1983; Miller et al., 1988). The segments were then run through the artifact detection and bad channel replacement tools again. This procedure allowed us to keep trials with eye blinks, although it did not remove the variance from blinks in non-eye channels.

For the current study, we focused on ERPs time-locked to the onset of the Flanker array and thus collapsed average waveforms across the cue condition, resulting in a final two averages per participant: congruent and incongruent (see below for more information). The average number of good segments (and accurate trials) out of a total of 96 trials for participants in the control tape condition was: congruent = 84 and incongruent = 82. The average number of good segments (and accurate trials) for participants in the meditation tape condition were: congruent = 88 and incongruent = 84.

## Results^4^

We again conducted independent samples *t*-tests to examine any differences between conditions (brief meditation vs. control tape) on variables including: age, gender, race, Big 5 Personality traits, and meditation experience (Table 2). Only one measure revealed a difference between groups, such that individuals randomly assigned to listen to the control tape reported higher levels of Conscientiousness (*M* = 0.30, *SD* = 1.01) than did those randomly assigned to listen to the meditation tape (*M* = −0.26, *SD* = 0.93; *t*_(52)_ = 2.10, *p* = 0.041). It is worth noting that applying a Bonferroni correction for multiple comparisons to these nine *t*-tests would require a *p*-value of 0.005 to achieve significance; thus, we are hesitant to report a significant group difference on any of the measures assessed.

**Table 2:** Means (SDs) for participants randomly assigned to listen to the meditation tape and the control tape in Study 2.

	Meditation tape	Control tape	*t*-statistic
*N*	29	27	
Age	19.41 (1.15)	19.63 (1.15)	0.70 (*p* = 0.49)
Gender (M = 0; F = 1)	0.38 (0.49)	0.59 (0.50)	1.60 (*p* = 0.12)
Race (White = 0; Non-White = 1)	0.48 (0.51)	0.69 (0.47)	1.58 (*p* = 0.12)
Big 5 Personality Traits (z-scored)			
Agreeableness	−0.14 (1.06)	0.16 (0.92)	1.08 (*p* = 0.28)
Conscientiousness	−0.26 (0.93)	0.30 (1.01)	2.10 (*p* = 0.04)
Extraversion	−0.09 (1.06)	0.10 (0.94)	0.69 (*p* = 0.49)
Neuroticism	0.12 (0.90)	−0.14 (1.11)	0.93 (*p* = 0.36)
Openness	0.05 (1.05)	−0.06 (0.96)	0.41 (*p* = 0.68)
Meditation experience*	1.05 (1.09)	0.67 (1.05)	1.20 (*p* = 0.24)

*An experimenter coded participants’ verbal descriptions of their experience with meditation on a scale from no experience (0) to regular practice (4).

To simplify analyses and focus on the effects of brief meditation on executive attentional processes (i.e., a replication and extension of Study 1), we limit analyses here to responses to congruent and incongruent trials, commonly recognized to be the best index of executive attentional control on the ANT (Fan et al., 2002). We used raw scores rather than difference scores (e.g., incongruent RTs—congruent RTs) for two reasons: (1) this allows for an investigation of overall differences in RTs as a function of other factors (i.e., neuroticism, meditation/control tape condition); and (2) if performance on the ANT is affected by meditation as in Study 1, using raw scores allows us to examine whether the effect is driven by incongruent or congruent trials.

### Behavioral Results

#### Reaction Times

As in Study 1, RTs for correct trials only were subjected to a 2 (condition: meditation, control) × 2 (trial type: congruent, incongruent) GLM, with the first factor manipulated between-participants and the second factor manipulated within-participants. The trial type main effect (*F*_(1,53)_ = 479.57, *p* < 0.001, $\eta_{p}^{2}$ = 0.90) indicated that participants were faster to respond on congruent trials (*M* = 498.98, *SE* = 7.70) than on incongruent trials (*M* = 596.35, *SE* = 8.41), replicating past results. More importantly, the condition main effect (*F*_(1,53)_ = 5.49, *p* = 0.023, $\eta_{p}^{2}$ = 0.09) showed that participants in the meditation condition were faster (*M* = 529.52 ms, *SE* = 10.86) than those in the control condition (*M* = 565.81, *SE* = 11.05), across trial types. This effect was not qualified by an interaction with trial type, suggesting that meditation may facilitate RTs in general.

#### Accuracy

In a second analysis, accuracy rates (i.e., proportions of correct trials) were subjected to a 2 (condition) × 2 (trial type) GLM. The trial type main effect (*F*_(1,53)_ = 67.52, *p* < 0.001, $\eta_{p}^{2}$ = 0.56) revealed that participants were more accurate on congruent trials (*M* = 0.99, *SE* = 0.002) than they were on incongruent (*M* = 0.94, *SE* = 0.007) trials. No other effects were significant.

#### Flanker Effect Scores

To further probe the effects of meditation on executive attention, we calculated difference scores in RTs on correct trials (incongruent—congruent) and accuracy (congruent—incongruent), separately. Two independent samples *t*-tests conducted on these difference scores revealed that meditation had no effect on either measure of executive attention for correct RTs, *t*_(53)_ = 0.36, *p* = 0.721, or for accuracy rates, *t*_(53)_ = 1.18, *p* = 0.244. Thus, the RT results provide support for Hypothesis 2a, and therefore constitute a conceptual replication of results from Study 1.

#### Moderation by Neuroticism

**Figure 5:**
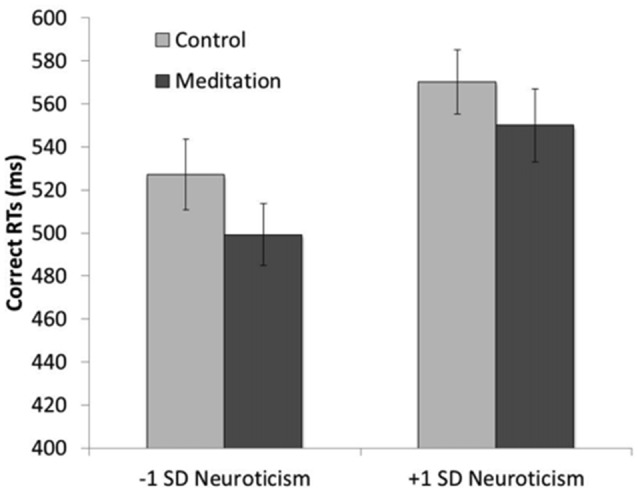
The interaction between neuroticism and condition in response times (RTs) on correct trials in Study 2. Individuals lower in neuroticism who listened to a meditation tape were faster on correct trials than were those who listened to a control tape. Individuals in the meditation group were faster on correct trials than were those in the control group; but only for those individuals lower in neuroticism.

As in Study 1, we conducted separate 2 (condition: meditation, control) × 2 (trial type: congruent, incongruent) × *z*-scored neuroticism GLMs on correct RTs and accuracy. For simplicity, only those effects concerning neuroticism are reported (i.e., the trial type main effect was significant in both analyses, as expected and reported above), and parameter estimates at 1 SD above and below the mean neuroticism score are used to understand the effects. The GLM conducted on correct RTs revealed a main effect of neuroticism (*F*_(1,48)_^5^ = 8.13, *p* = 0.006, $\eta_{p}^{2}$ = 0.15), such that participants higher in neuroticism (+1 SD) were slower (*M* = 568.96 ms, *SE* = 11.01) than were participants lower in neuroticism (−1 SD; *M* = 524.39, *SE* = 10.93). Although the neuroticism × condition interaction did not reach significance (*F*_(1,48)_ = 0.36, *p* = 0.55, $\eta_{p}^{2}$ = 0.01), the main effect of condition was weakened when controlling for neuroticism (*F*_(1,48)_ = 3.29, *p* = 0.076, $\eta_{p}^{2}$ = 0.06). This pattern suggests that meditation did have a different effect on correct RTs for individuals lower and higher in neuroticism; therefore, we examined pairwise comparisons for the neuroticism x condition interaction. Participants higher in neuroticism showed no effect of meditation on overall correct RTs, such that those in the control condition (*M* = 578.21 ms, *SE* = 14.23) were not significantly slower to respond than those in the meditation condition (*M* = 559.70, *SE* = 16.77; *p* = 0.41). However, participants lower in neuroticism were marginally faster to respond in the meditation condition (*M* = 505.74, *SE* = 14.68) than were those in the control condition (*M* = 543.03, *SE* = 16.20; *p* = 0.094; Figure 5). In sum, meditation did reduce RTs, but more so for individuals lower in neuroticism.

A similar GLM conducted on accuracy rates merely revealed the main effect of trial type reported above; no effects involving neuroticism were significant. Thus, the RT results provide support for Hypothesis 2b, and therefore constitute a conceptual replication of results from Study 1.

### ERP Results

**Figure 6:**
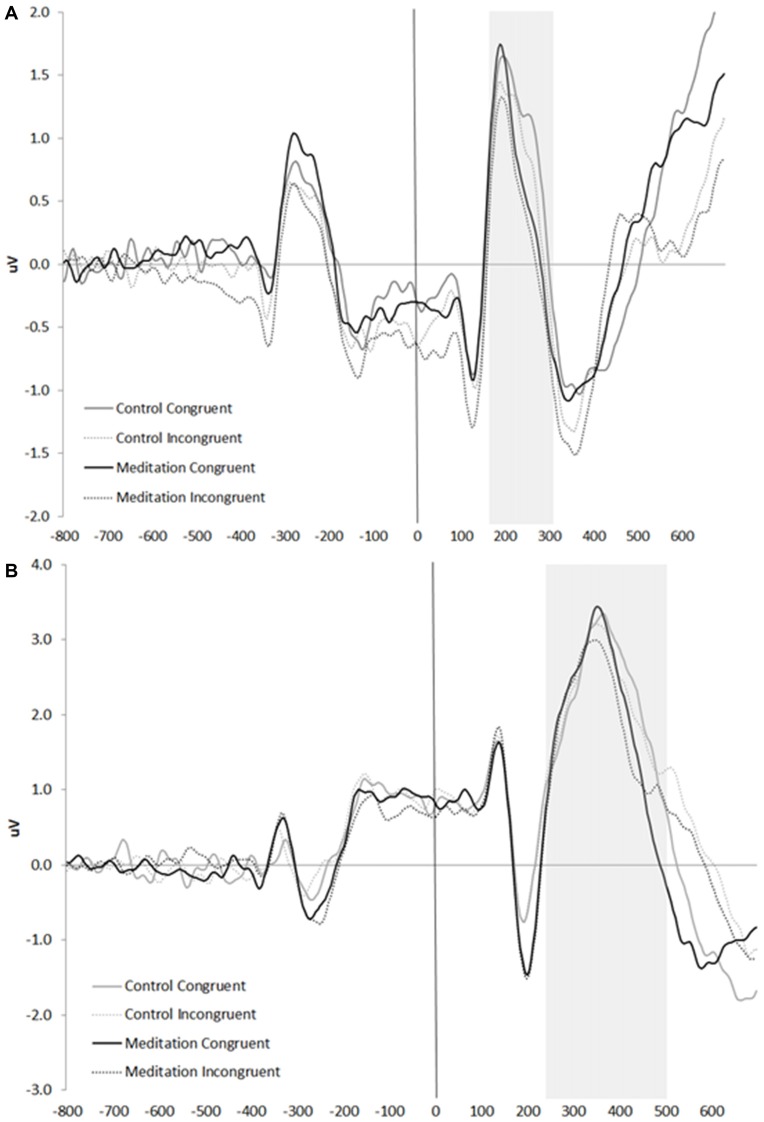
Grand averaged waveforms at **(A)** Fz and **(B)** Pz. The solid vertical line indicates the onset of the Flanker stimulus; the gray boxes indicate the time window analyzed for the **(A)** N2 and **(B)** P3b.

We focused on two event-related brain potentials in Study 2, based on previous research (Kopp et al., 1996a, b; Heil et al., 2000; Nieuwenhuis et al., 2003; Hietanen et al., 2008; Neuhaus et al., 2010). Measurement windows were determined based on these earlier studies. First, we measured the simple peak amplitude of the N2, a negative-going component occurring at approximately 200 ms post-stimulus, as the most negative value occurring at site Fz (midline frontal) between 100 ms and 300 ms post-Flanker array. Second, we defined the mean amplitude of the P3b, a positive-going component occurring between 250 ms and 500 ms post-stimulus, as the average voltage during that time window at site Pz (midline parietal; Luck, 2005). Grand averaged waveforms are presented in Figure 6.

### N2 Amplitudes

**Figure 7:**
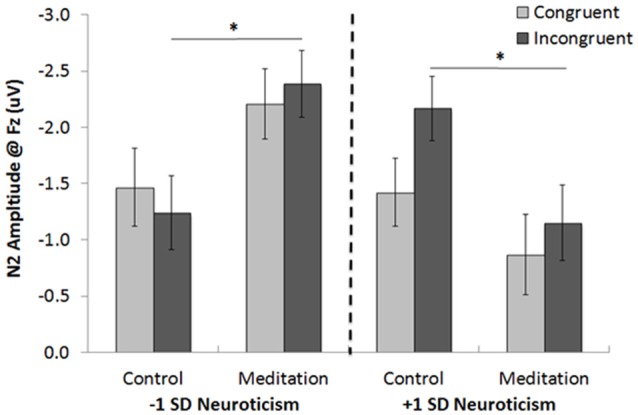
The 3-way interaction between neuroticism, meditation/control tape condition and trial type in Study 2 on N2 amplitudes at Fz (Note: negativity is plotted up for ease of interpretation; higher N2 amplitudes will thus be presented as taller bars). *p < 0.05.

A 2 (condition: control, meditation) × 2 (trial type: congruent, incongruent) × *z*-scored neuroticism GLM was conducted on N2 amplitudes at Fz. Consistent with previous research, the trial main effect (*F*_(1,48)_ = 3.49, one-tailed *p* = 0.03, $\eta_{p}^{2}$ = 0.07) revealed larger amplitude N2 s for incongruent (*M* = −1.74, *SE* = 0.15) than for congruent (*M* = −1.49, *SE* = 0.16) trials. This effect, however, was qualified by a number of higher order interactions. First, the trial × neuroticism interaction (*F*_(1,48)_ = 4.08, *p* < 0.05, $\eta_{p}^{2}$ = 0.08) showed that the N2 amplitude difference between incongruent (*M* = −1.66, *SE* = 0.22) and congruent (*M* = −1.15, *SE* = 0.24) trials was significant for individuals higher in neuroticism (*p* = 0.009); but not for those individuals lower in neuroticism (*M*_incongruent_ = −1.81, *SE* = 0.22; *M*_congruent_ = −1.84, *SE* = 0.23, *p* = 0.90). Neuroticism also interacted with condition (*F*_(1,48)_ = 8.56, *p* = 0.005, $\eta_{p}^{2}$ = 0.15), such that—across trial types—individuals lower in neuroticism who listened to the meditation tape showed enhanced N2 amplitudes (*M* = −2.30, *SE* = 0.28) compared to those who listened to the control tape (*M* = −1.35, *SE* = 0.31, *p* = 0.027); whereas individuals higher in neuroticism who listened to the meditation tape showed marginally reduced N2 amplitudes (*M* = −1.01, *SE* = 0.32) compared to those who listened to the control tape (*M* = −1.80, *SE* = 0.27, *p* = 0.065). Finally, the 3-way condition × trial type × neuroticism interaction was marginally significant (*F*_(1,48)_ = 2.70, *p* = 0.107, $\eta_{p}^{2}$ = 0.05). Because all three factors were implicated in multiple interactions and because of our central interest in the 3-way interaction, we conducted pairwise tests to break it down (Figure 7). As predicted, individuals lower in neuroticism who listened to the meditation tape showed enhanced N2 amplitudes to incongruent trials (*M* = −2.39, *SE* = 0.30) compared to those who listened to the control tape (*M* = −1.24, *SE* = 0.33; *p* = 0.012). On the contrary, individuals higher in neuroticism who listened to the meditation tape showed reduced N2 amplitudes to incongruent trials (*M* = −1.15, *SE* = 0.34) compared to those who listened to the control tape (*M* = −2.17, *SE* = 0.29; *p* = 0.026). Thus, neuroticism and meditation interacted to impact N2 amplitudes on incongruent trials, indicating differential sensitivity to conflict.

#### P3b Areas

**Figure 8:**
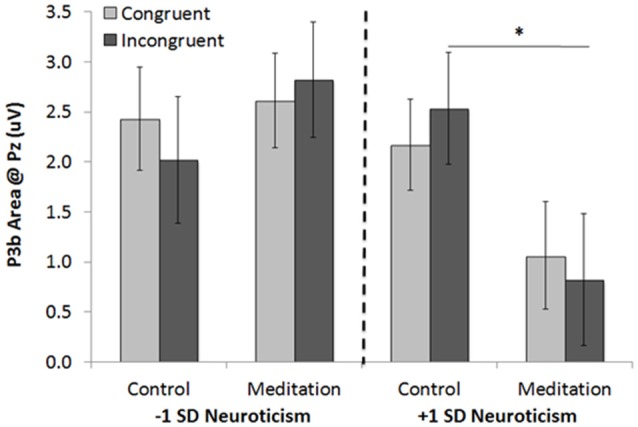
The 3-way interaction between neuroticism, meditation/control tape condition, and trial type in Study 2 on P3b areas at Pz (Note: positivity is plotted up for ease of interpretation; higher P3b amplitudes will thus be presented as taller bars). *p < 0.05.

An identical 2 (condition: control, meditation) × 2 (trial type: congruent, incongruent) × *z*-scored neuroticism GLM was conducted on P3b areas at Pz. The only significant effect to emerge was the 3-way interaction (*F*_(1,48)_ = 4.70, *p* = 0.035, $\eta_{p}^{2}$ = 0.09; Figure 8). Pairwise tests showed that individuals lower in neuroticism who listened to the meditation tape exhibited no differences in the P3b (*M*_congruent_ = 2.61, *SE* = 0.47; *M*_incongruent_ = 2.82, *SE* = 0.58) compared to those who listened to the control tape (*M*_congruent_ = 2.43, *SE* = 0.52, *p* = 80; *M*_incongruent_ = 2.02, *SE* = 0.64, *p* = 0.36). Individuals higher in neuroticism who listened to the meditation tape, however, exhibited a *marginally* *reduced* P3b to incongruent trials (*M* = 0.82, *SE* = 0.66) as compared to those who listened to the control tape (*M* = 2.54, *SE* = 0.56; *p* = 0.054); there were no differences in the P3b for congruent trials between those who listened to the meditation tape (*M* = 1.06, *SE* = 0.54) and those who listened to the control tape (*M* = 2.17, *SE* = 0.45, *p* = 0.12). In sum, results for the P3b parallel those for the N2 such that in both cases, individuals higher in neuroticism showed reductions on neural indices of attentional allocation on incongruent trials after listening to a brief mindfulness meditation tape. Results for individuals lower in neuroticism, however, differed for the N2 (which indicated *improvements* on incongruent trials after listening to a meditation tape) and the P3b (which showed no differences in for the meditation vs. control tape conditions). Interestingly, results from both the N2 and the P3b only partially supported Hypothesis 3. The N2 results supported the hypothesis as individuals lower in neuroticism exhibited increased attention, while those higher in neuroticism did not. However, the P3b results did not show a benefit for individuals lower in neuroticism, and suggested a marginal reduction in individuals high in neuroticism.

#### Correlations Between N2 and P3b

Finally, we conducted correlations to investigate relationships between the N2 and P3b components of the ERP. This analysis was motivated by a number of factors. First, the patterns of data observed for the N2 and P3b components in the current study were very similar. Second, this is not surprising, given that much research on cognitive control (e.g., using flanker tasks, go/no-go tasks, or the ANT) focuses on these two components, their role in cognitive control, and their relationship to each other (e.g., Patel and Azzam, 2005; Rietdijk et al., 2014; Groom and Cragg, 2015). Indeed, some researchers even refer to these components as the N2/P3 complex, emphasizing their association (Azizian et al., 2006). Thus, we examined correlations between the N2 and P3b, as well as the impact of meditation on these relationships.

First, the N2 was significantly correlated with the P3b for both congruent (*r*_(53)_ = −0.50, *p* < 0.001) and incongruent (*r*_(53)_ = −0.43, *p* = 0.001) trials. Because of the polarity of these components, this inverse relationship actually indicates a positive relationship, such that larger (i.e., more negative) N2 amplitudes were associated with larger (i.e., more positive) P3b areas. Second, we conducted correlations separately for the meditation and control conditions. For participants who listened to the control tape, the N2 was not significantly correlated with the P3b for either congruent (*r*_(24)_ = −0.38, *p* > 0.05) or incongruent (*r*_(24)_ = −0.30, *p* > 0.05) trials. Importantly, the N2 was correlated with the P3b for both congruent (*r*_(27)_ = −0.64, *p* < 0.001) and incongruent (*r*_(27)_ = −0.60, *p* = 0.001) trials for participants who listened to the meditation tape. Thus, after listening to the meditation tape, larger N2s (indicating enhanced attentional control) were associated with larger P3bs (indicating enhanced attention allocation); this pattern was not significant for participants who listened to the control tape.

## Discussion

Individuals who completed a brief meditation had faster correct RTs on the ANT, regardless of trial type, than did those in the control condition, especially when they were relatively lower in neuroticism (Hypothesis 2a and 2b). N2 amplitudes replicated past results, as they were larger to incongruent than to congruent trials. The N2 is implicated in conflict detection and executive attention; larger amplitude N2s are often observed to incongruent (conflict) than to congruent (no conflict) trials on multiple paradigms (e.g., Flanker, Stroop), and our results are consistent with such past research (Kopp et al., 1996a, b; Heil et al., 2000). However, this finding was qualified by multiple interactions, including the 3-way interaction between trial type, meditation/control tape condition, and neuroticism. Compared to the control condition, meditation was associated with larger N2 amplitudes to incongruent trials, consistent with the idea that executive attention is enhanced following a brief meditation—but only for individuals lower in neuroticism. Interestingly, the pattern was reversed for individuals higher in neuroticism; for them, meditation was associated with smaller N2 amplitudes to incongruent trials. Given the known role of the N2 in shifting attention, response competition, and executive function (Kopp et al., 1996b; Heil et al., 2000; Nieuwenhuis et al., 2003; Hietanen et al., 2008), these results suggest that such functions are improved when individuals who are low in neuroticism undergo a brief meditation intervention. Individuals higher in neuroticism, on the other hand, might show a detriment in allocation of attention toward more difficult (i.e., incongruent) trials after listening to a meditation tape. Although this was a marginal effect, it could suggest that perhaps the meditation instructions interfered with their ability to efficiently shift attention when needed (Hypothesis 3).

Results for the P3b also showed an interaction between tape condition, trial type, and neuroticism, such that individuals higher in neuroticism who listened to the meditation tape exhibited a marginally reduced P3b on incongruent trials as compared to those who listened to the control tape. Although these findings are similar to those for the N2, the difference between them is informative for our understanding of how meditation may impact neural processes underlying attention. Specifically, individuals higher in neuroticism fail to show an enhanced N2 after listening to a meditation tape, suggesting that their attention to conflict and/or competing responses is not improved by meditation; and they show a marginally reduced P3b after listening to a meditation tape, suggesting that their allocation of attention toward incongruent trials may be relatively reduced to a small degree. Together, these findings indicate that individuals higher in neuroticism may not benefit from meditation on a task requiring focused executive attention (e.g., Flanker, ANT) after listening to a meditation tape (Hypothesis 3), consistent with behavioral results from both Study 1 and Study 2. Finally, the N2 was correlated with the P3b, primarily for individuals who listened to the meditation tape, indicating that meditation may synergistically impact both earlier control of attention and later attention allocation.

## General Discussion

Mindfulness meditation practice is known to affect various psychological outcomes, including cognitive performance and attention. Across two studies, we tested the boundary conditions of brief mindfulness meditation, and showed that even a very small “dose” can have beneficial effects in individuals with very little or no practice—especially in individuals lower in neuroticism. Although the two studies represent conceptual replications of the beneficial effect of brief meditation, results from Study 2 diverge somewhat from those from Study 1. Specifically, results from Study 2 showed that brief meditation improves RTs on correct trials instead of accuracy, and that this performance boost may generalize to all trial types (i.e., congruent, incongruent, neutral) rather than being specific to those requiring strong attentional control (i.e., incongruent trials, as in Study 1). However, considering the differences between the Flanker task used in Study 1 and the ANT used in Study 2 may shed light on this apparent divergence in findings. Most critical is the absence of intertrial intervals in Study 1; as soon as a response was made on one trial, a new trial began. This had the explicit purpose of decreasing the length of the experiment for participants, but may have increased anxiety, depleted cognitive resources, and increased the need for speeded responses (although it did not negatively impact accuracy rates, as can be seen by a comparison of accuracy rates in Study 1 and Study 2). Indeed, RTs in Study 2 were generally slower than in Study 1, suggesting that participants in Study 1 were forced to respond more quickly. Notably, individuals who had listened to the meditation tape had shorter RTs in Study 2 (regardless of condition), suggesting that they were more focused and able to perform the task more quickly.

Of primary interest is that a brief meditation period did affect performance in both studies: when under time pressure in Study 1, participants in the meditation condition showed increased attentional control, as exhibited by better performance on incongruent trials; and when given short breaks between trials in Study 2, participants in the meditation condition showed faster correct RTs overall, regardless of trial type, consistent with the conclusion that they were better able to focus and respond (correctly) more quickly than those in the control condition. Thus, a brief guided meditation may improve executive attention, but the manifestation of that improvement may depend strongly on the task being performed. This conclusion is not surprising, given the diversity of findings in the literature on the role of meditation (whether long-term or brief, in practitioners or novices) on attention, and suggests that more careful delineation of task requirements, underlying processes, and performance measures may be required to further our understanding of this complex relationship and its boundary conditions.

Importantly, across both studies, neuroticism moderated the effects of brief meditation on attentional processes in novices. Results from both studies indicated that individuals lower in neuroticism exhibited performance boosts following meditation; whereas those higher in neuroticism performed equally well in the control and meditation conditions. In addition, individuals lower in neuroticism who listened to the meditation tape showed increased N2 amplitudes on incongruent trials, suggesting an improvement in detecting conflict (although this was not mirrored in the behavioral data). Neuroticism is characterized by anxiety, high negative affect, worry, and moodiness, and is often associated with self-consciousness, difficulty to control urges, and weakened self-regulation (e.g., Robinson, 2007). Thus, it seems as though individuals higher in neuroticism may have difficulty reaping the benefits of a brief meditation, possibly due to increased self-awareness and anxiety. The finding that neuroticism moderates the effects of meditation on attention is particularly important because it may explain why prior studies of brief meditation failed to find an effect on cognitive functions (Larson et al., 2013; Johnson et al., 2015). In future studies, measuring and controlling for individual differences in neuroticism may be necessary for uncovering the effects of brief sessions of mindfulness meditation on cognition.

Previous research has focused on the reduction of neuroticism, anxiety and stress due to meditation and less on personality predictors (e.g., neuroticism) of response to meditation. For example, Williams et al. (1976) found that males who practiced transcendental meditation (i.e., a self-selected group) were more neurotic than the general population, but that they also became less neurotic over the course of a 6-month period of study and that decreases in neuroticism were directly associated with frequency of meditation. Similarly, Lane et al. (2007) found that neuroticism moderated treatment effects in a group of individuals who completed training in meditation, such that individuals higher in neuroticism at baseline showed greater decreases in negative mood, perceived stress and anxiety over the course of training. Thus, neuroticism may have a differential impact on consequences of long-term meditation training. Less is known about its ability to predict who will benefit from the practice of meditation.

In an early review of the literature, Delmonte (1985) argued that prospective meditators often report higher-than-average anxiety levels, and that anxiety predicts lower frequency of practice. Ironically, Delmonte (1985) also reported that meditation reliably decreases levels of anxiety over the course of practice, a finding that has since been replicated widely (e.g., Goldin and Gross, 2010; Piet et al., 2012; Vøllestad et al., 2012). Furthermore, studies have shown that regular meditators tend to be lower in trait neuroticism (Delmonte, 1985; Leung and Singhal, 2004). Thus, greater neuroticism may drive individuals to engage in meditation, while also negatively impacting the frequency of practice, and perhaps preventing any early beneficial effects. If they do persist in meditation, however, these individuals often show decreases in their anxiety and negative affect.

Specifically, our results suggest that trait neuroticism may reduce the efficacy of short, guided meditation; individuals high in anxiety may not be able to relax and follow the instructions presented during their first brief meditation, thus preventing them from reaping the benefits of this intervention. This finding has strong implications for the field, as it suggests that the very population thought to benefit most from meditation (i.e., individuals high in anxiety and neuroticism) may have difficulty initially engaging in the practice. As meditation becomes more frequently prescribed as part of a holistic treatment for mental health disorders often associated with high neuroticism, including depression, phobia, and other anxiety disorders, clinicians would benefit from an understanding of the difficulties individuals high in neuroticism face in both learning and persisting in the practice of mindfulness meditation.

In sum, our results suggest that even in novices, one brief 10-min audio-guided mindfulness meditation instruction period improves attention. The observed performance improvements varied as a function of the cognitive demands placed on the individual. When time pressure was applied, participants in the meditation condition exhibited a boost in accuracy reflecting increased attentional control (Study 1). When the task was more complex but less temporally constrained, participants in the meditation condition were faster to respond correctly, regardless of the presence or absence of distracting stimuli (Study 2). Importantly, these effects were strongest for individuals lower in neuroticism, indicating that personality may impact the ability to reap the benefits of brief meditation. ERP results suggest two possible mechanisms underlying the effects of meditation on attention: an improved ability to control attention toward conflicting stimuli, as evidenced by larger N2 amplitudes following meditation; and more efficient attention allocation, as evidenced by maintained P3b areas following meditation—although both of these effects were moderated by individual differences in neuroticism.

## Limitations

By investigating a very brief period of mindfulness meditation in meditation-naïve participants, this study uniquely adds to our understanding of dose effects of meditation practice. Nevertheless, there remain a number of limitations and unanswered questions. One is the role of neuroticism in moderating the efficacy of brief meditation interventions. We suggest that neuroticism, a trait often associated with anxiety and self-consciousness, may directly impact the ability of individuals high in neuroticism to engage with mindfulness instructions. Another possibility is that neuroticism may simply be a marker for a lack of a trait mindfulness. However, there are a few problems with this interpretation: first, the relationship between neuroticism and dispositional mindfulness is complicated, and is not simply inverse. Feldman et al. (2010), among others, have found a correlation between neuroticism and dispositional mindfulness. But they have also found that these two factors are not redundant—each independently predicted trait anger and depressive symptomology. Furthermore, the two factors interacted in their effects on a separate factor, suggesting that they moderate each other (which further supports the conclusion that they are not redundant). Second, we manipulated mindfulness in participants assigned to the meditation condition; we did not simply examine the relationship between dispositional mindfulness and executive attention. Thus, although we acknowledge that the relationship between neuroticism and dispositional mindfulness is one that researchers should consider further in future studies, it is unlikely to account for the current data.

Furthermore, the term “mindfulness” itself may represent multiple practices and processes (e.g., van Dam et al., 2018), and it is possible that this initial mindfulness meditation period is only partly related to the mindfulness state that may be generated by experienced meditators. Nevertheless, we modeled our instructions after typical definitions used by Kabat-Zinn and others (e.g., Bishop et al., 2004; Kabat-Zinn, 2017) as embodied in the foundational meditations in MBSR. Specifically, these components include attention to the present moment that is characterized by an open, curious and accepting attitude. As such, this mindfulness meditation period may not represent the deeper mindfulness states associated with long-term training, but rather one’s initial contact with a mindful state, as might occur during the initial meditation in an MBSR course.

It is also worth noting that this is a preliminary study, and the first to our knowledge to test the effects of one’s first encounter with mindfulness meditation instructions, as one might do when beginning an MBSR course. Thus, we were not yet trying to dismantle the effects of different components of mindfulness in this study. Rather, we examined whether listening to a tape containing 10 min of mindfulness meditation instructions (derived from those given in MBSR courses) can have an effect on attention. As such, we chose a control condition that was matched on number of words, word frequencies, voice, cadence, and length, but that differed in content. Future work can further dismantle which component of the instructions led to the observed differences (shown here across two independent samples) by generating additional controls. However, given that this is the first study of its kind to our knowledge, we believe the current control condition is sufficient to show preliminary differences between mindfulness meditation instructions and the control tape.

In addition, it is also worth noting that, just as intensive or immersive forms of training in mindfulness meditation have their limitations, brief interventions might also have their limitations. Whereas participation in a days-, weeks-, or months-long mindfulness training program is limited by the time and resources involved, in addition to the motivation necessary for an individual to do so, a brief 10-min audio-guided meditation requires little to no motivation, time, or money. However, while long-term meditation training and practice has shown to reap broad and lasting benefits in cognitive abilities (Chiesa et al., 2011), psychological health (Keng et al., 2011) and even physical health (Grossman et al., 2004), the effects of brief meditation interventions on meditation-naïve individuals may be transient and/or fleeting and may not impact well-being or transfer to everyday life. Thus, as noted previously, future studies should continue to explore dose effects of meditation practice, as well as the timeline along which meditation interventions affect cognitive processes and mental and physical health.

## Conclusion

Ultimately, although much remains to be studied, the current studies expand our understanding of the initial effects of brief meditation, and suggest that brief meditation impacts attention even in novice practitioners—an effect that was revealed when controlling for neuroticism. In addition, it is worth noting that the current studies are not just useful for unlocking the wellness benefits of meditation; they may also be useful for the psychological study of attention in general. Indeed, by understanding how meditation affects certain components and neural mechanisms of attention, researchers may better understand the processes underlying this complex, multifaceted cognitive ability. Thus, the findings have theoretical, clinical and methodological implications.

## Author Contributions

CN and DC both designed the study. DC collected the data and CN performed the analyses. CN wrote the manuscript based in part on a thesis submitted by DC. All authors contributed to the clarity and accuracy of writing. RH and HK developed, tested and normed the audio tapes and assisted with the literature review.

## Conflict of Interest Statement

The authors declare that the research was conducted in the absence of any commercial or financial relationships that could be construed as a potential conflict of interest.

## Footnotes

^1^We do not address here those studies that have focused solely on the effects of mindfulness meditation on affect or emotional processes (e.g., Levitt et al., 2004; Broderick, 2005; Arch and Craske, 2005; Erisman and Roemer, 2010; Feldman et al., 2010; Alberts and Thewissen, 2011; Tan et al., 2014). Nor do we summarize studies using brief meditation in clinical samples (e.g., Lee and Orsillo, 2014; Tonelli and Wachholtz, 2014).

^2^A Bonferroni correction was applied to all pairwise tests throughout to correct for multiple comparisons.

^3^www.egi.com

^4^As in Study 1, a Bonferroni correction was applied to all pairwise tests throughout to correct for multiple comparisons.

^5^Degrees of freedom differ from initial analyses, as neuroticism scores for five participants were either not collected due to time constraints or were lost due to program error.
