# Corrigendum: Brief Mindfulness Meditation Improves Attention in Novices: Evidence From ERPs and Moderation by Neuroticism

**DOI:** 10.3389/fnhum.2018.00342

**Published:** 2018-09-05

**Authors:** Catherine J. Norris, Daniel Creem, Reuben Hendler, Hedy Kober

**Affiliations:** ^1^Department of Psychology, Swarthmore College, Swarthmore, PA, United States; ^2^Psychiatry Department, Massachusetts General Hospital, Boston, MA, United States; ^3^Departments of Psychiatry and Psychology, Yale University, New Haven, CT, United States

**Keywords:** mindfulness meditation, neuroticism, attention, flanker, ANT, N2, P3b

In the published article, there was an error regarding the affiliations for Hedy Kober. As well as having affiliation(s) Department of Psychology, Yale University, New Haven, CT, United States they should also have Department of Psychiatry, Yale University, New Haven, CT, United States. The authors apologize for this error and state that this does not change the scientific conclusions of the article in any way.

The original article has been updated.

## Conflict of interest statement

The authors declare that the research was conducted in the absence of any commercial or financial relationships that could be construed as a potential conflict of interest.

